# The quality and reliability of short videos about myocardial infarction on TikTok: a cross-sectional study

**DOI:** 10.3389/fpubh.2026.1751884

**Published:** 2026-03-12

**Authors:** Shudi Li, Menghe Zhang, Yaoyao Zuo, Lin Zhou, Zhenhai Sun, Tailong Lv, Huidan Xie, Shouqiang Chen

**Affiliations:** 1 Shandong University of Traditional Chinese Medicine, Jinan, Shandong, China; 2 The Second Affiliated Hospital of Shandong University of Traditional Chinese Medicine, Jinan, Shandong, China

**Keywords:** myocardial infarction, reliability, social media, TikTok, video quality

## Abstract

**Introduction:**

Myocardial infarction (MI) is one of the major diseases affecting human health and life, characterized by its acute onset and high mortality rate. Social media platforms like TikTok are playing an increasingly important role in disease education and prevention. This study evaluated the quality of MI-related science education videos on TikTok and examined the relationship between video content quality and user engagement.

**Methods:**

Video quality was assessed using the Global Quality Scale (GQS), Journal of the American Medical Association (JAMA) benchmark standards, and the modified DISCERN (mDISCERN) tool. Video duration and engagement metrics (likes, comments, shares, and collections) were recorded. Spearman’s correlation and linear regression analyses were employed to examine the relationship between video quality and engagement.

**Results:**

After screening the videos, a total of 270 videos were analyzed. Collectively, these videos garnered 35,913,507 likes, 12,745,256 shares, 8,072,837 collections, and 1,306,276 comments. Most videos (84.1%, *n*=227) were uploaded by healthcare professionals, predominantly Western medicine practitioners (83.7%, 190/227). The highest proportion of videos scored 3 on the GQS scale (47.0%, 127/270). The highest proportion of videos scored 2 on the JAMA scale, accounting for 81.5% (220/270). The highest proportion of videos scored 3 on the mDISCERN scale, accounting for 45.9% (124/270). Video duration showed a significant positive correlation with both GQS and mDISCERN scores. The number of bookmarks correlated positively with both JAMA and mDISCERN scores.

**Discussion:**

As a widely used video dissemination platform, TikTok provides users with a positive experience, with the overall quality of MI-related short videos remaining at a moderate level. Analysis indicates that extending video duration and appropriately citing references and sources can further enhance video quality and reliability, offering users more comprehensive knowledge and improved disease prevention outcomes.

## 1 Background

Myocardial Infarction (MI) is a condition caused by coronary artery occlusion that interrupts forward blood flow, leading to myocardial ischemia and hypoxia. This manifests as chest tightness and crushing pain in the precordial region. Severe cases may even result in arrhythmia, cardiac arrest, or sudden death (1–4). MI ranks among the most significant threats to human health, characterized by acute onset, high mortality rates, and poor prognosis (5, 6). With China’s rapid economic development, the prevalence of hypertension, diabetes, and hyperlipidemia has surged, compounded by mounting work and life pressures, leading to a growing incidence of MI (7). Research indicates cardiovascular disease is the leading cause of death among both urban and rural residents in China, accounting for 43.56% of urban deaths and 45.91% of rural deaths, with approximately 4 million annual fatalities (8–10). Over 70% of MI deaths occur outside hospitals, making precise mortality statistics challenging. Estimates suggest annual acute MI deaths in China range between 600,000 and 1,000,000 (11). Numerous MI patients die due to lack of timely treatment. Therefore, enhancing public awareness and early recognition of MI in China is particularly crucial.

Short video platforms play an increasingly vital role in health communication, disease education, and user access to medical information (12). As one of the world’s most popular short video social media platforms, TikTok boasts massive traffic, has gained a broad user base, and is gradually becoming an important platform for medical science communication (13). This platform effectively presents diverse information on healthcare, wellness, and health preservation through integrated videos, animations, music, and text, covering a broad spectrum of topics (14). It has become the world’s most downloaded short video platform, accumulating 24.8 billion downloads by 2025. With advancements in artificial intelligence and growing public concern about health issues, an increasing number of users now turn to short videos to learn about medical conditions before seeking professional care. TikTok has thus become a vital source for accessing health information (15). Previous studies on short videos related to hypertension, ankle sprains, gastroesophageal reflux, testicular pain, and testicular cancer have shown that the quality and reliability of these videos vary significantly (16–20).

Currently, science-popularization videos about MI are proliferating, and public awareness of this condition is steadily increasing. However, research on the quality and reliability of MI-related videos remains scarce. To evaluate the quality and reliability of such content, we drew upon previous studies assessing video quality and reliability (21). We scored MI-related short videos on TikTok using the Global Quality Scale (GQS), the Journal of the American Medical Association (JAMA) benchmark criteria, and the modified DISCERN (mDISCERN) tool. We also analyzed the relationship between video quality and key engagement metrics which including likes, shares, collections, comments, and video duration for these TikTok videos. Through this study, we aim to understand the quality and reliability of MI-related short videos on TikTok, enhance the video quality of medical science communicators, and improve public understanding of symptoms before and during MI onset, as well as medical treatment strategies. This may help reduce mortality and disability rates among MI patients and improve quality of life (22, 23).

## 2 Materials and methods

### 2.1 Extracting basic information

China’s TikTok search engine exhibits the characteristic of short keywords covering longer terms. Searching with short keywords can retrieve videos containing the full, longer keyword phrases. In the Chinese context, “心肌梗死” is the standard medical term corresponding to “myocardial infarction” in English, while “心梗” is its colloquial abbreviation, corresponding to “heart attack.” Using the short keywords “myocardial infarction” and “heart attack” yields videos covering various related topics containing the long keywords “myocardial infarction, acute myocardial infarction, ST-elevation myocardial infarction, and non-ST-elevation myocardial infarction. Therefore, we conducted a systematic search on TikTok using “myocardial infarction” and “heart attack” as keywords on August 7, 2025, targeting videos related to MI published before August 7, 2025. To minimize personalized recommendations and biases influenced by individual preferences and search history, our data collection was performed without user account authentication. Videos were not restricted by publication date and were sorted comprehensively. A total of 315 MI-related videos were retrieved. Engagement metrics for the short videos which including likes, collections, shares, comments along with video links were exported to an Excel spreadsheet. Collections mean the user stores their favorite video, audio, or other content in their personal account for later viewing. Likes signify user approval and appreciation for a video’s content. Comments represent user interactions with creators and other users, sharing viewing experiences and thoughts. Shares denote users distributing preferred videos to other users or platforms.

After viewing all videos, we applied the following filters: (1) Excluded videos unrelated to MI; (2) Removed invalid links; (3) Exclude videos posted within the last 7 days (i.e., August 1, 2025–August 7, 2025), as engagement metrics like likes, shares, comments, and collections for recent content are not yet stable; (4) Exclude non-Mandarin-language videos; (5) Remove duplicate videos.

### 2.2 Quality assessment

To ensure rigor and independence, a double-blind evaluation process was employed. Two independent researchers (MZ and SL) assessed each video separately using the GQS, JAMA, and mDISCERN scoring scales without communication, recording each video’s scale scores. The GQS uses a 1–5 point scale to reflect content quality. The JAMA scale assessed four dimensions: attribution, timeliness, content validity, and disclosure compliance, scored from 0 to 4 (24). The mDISCERN scale primarily evaluated five aspects: clarity, relevance, traceability, robustness, and fairness, scored from 0 to 5 in ascending order (25, 26). Final scores were determined by comparing the two evaluators’ ratings. In cases of disagreement, a third researcher (YZ) was invited to assess the video quality, with the final score being the rating agreed upon by two evaluators.

### 2.3 Statistical analysis

Statistical analysis was performed using SPSS version 26.0. Continuous variables following a normal distribution were described using mean and standard deviation, while non-normally distributed continuous variables were characterized by median and interquartile range. Categorical variables were presented as frequencies and percentages. Continuous variables meeting normality assumptions were analyzed using independent samples *t*-tests; non-normally distributed continuous variables underwent nonparametric rank-sum tests. Spearman correlation analysis and linear regression analysis assessed inter-variable correlations. *p* < 0.05 was considered statistically significant, while *p* < 0.01 indicated substantial statistical significance.

## 3 Results

**Figure 1:**
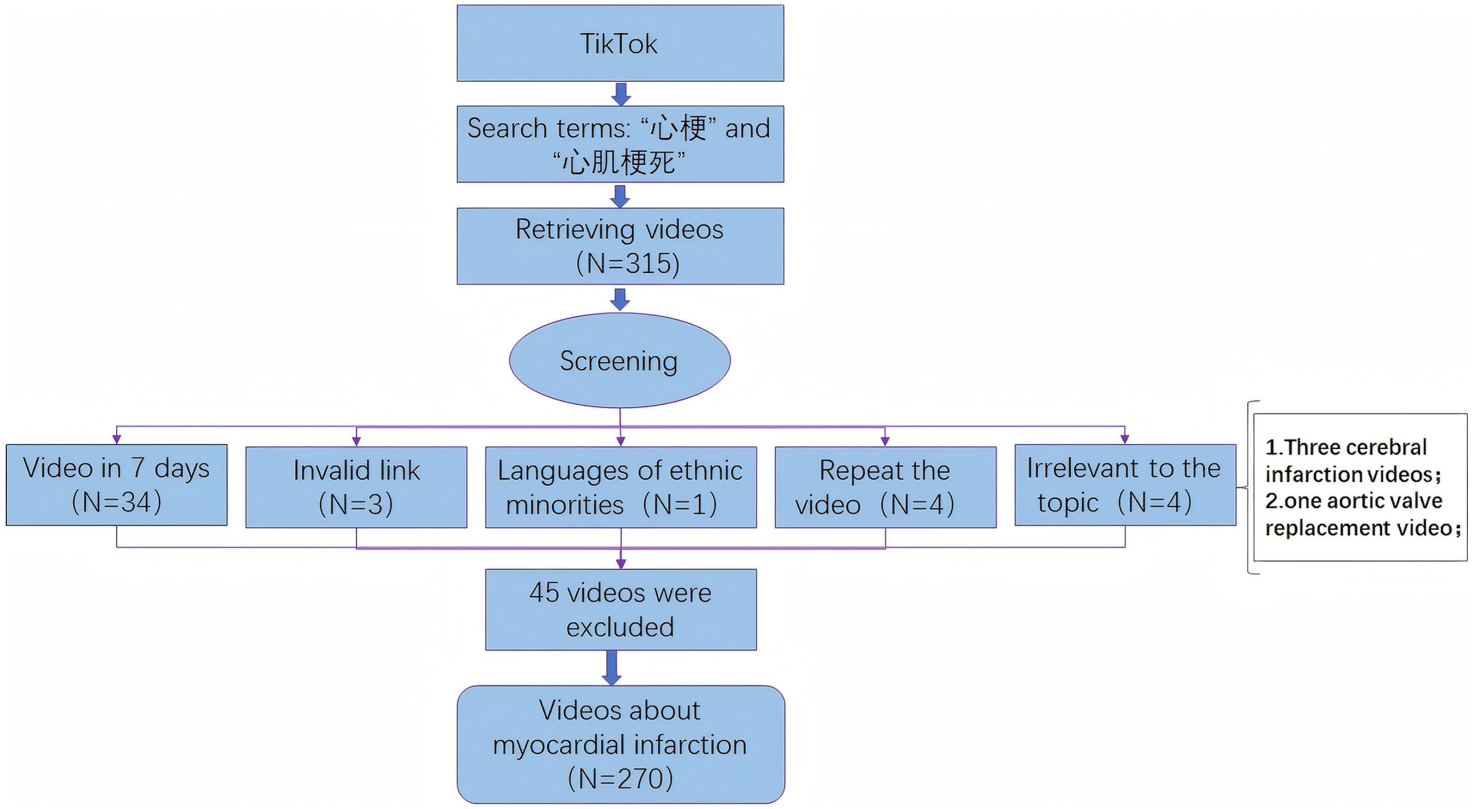
Video screening flowchart.

As shown in Figure 1, we searched TikTok using the keywords “myocardial infarction” and “heart attack,” yielding a total of 315 related videos. After screening, we excluded 34 videos posted within the past week; removed 3 invalid links; excluded 1 video in a minority language; removed 3 duplicate videos; and filtered out 4 videos unrelated to MI (three featuring cerebral infarction and one on aortic valve replacement). This yielded 270 relevant MI videos. These serve as valuable resources for public education on MI and for learning about daily medical care related to the condition.

### 3.1 Characteristics of MI videos

Analysis reveals that 270 MI-related short videos on TikTok garnered a total of 35,913,507 likes, 1,306,276 comments, 8,072,837 collections, and 12,745,256 shares. Chronologically, the earliest MI video on TikTok originated from the official media account “Yunshang Nanyang” on November 29, 2020. Titled “Approximately 1 Million Sudden Deaths from Heart Attacks Occur Annually in China, Linked to These 4 Unhealthy Habits!,” it served as a public science video on MI. This video provides a detailed explanation of the annual number of sudden deaths from MI in China, its symptoms, risk factors, and emergency treatment methods, offering an intuitive and comprehensive science education resource for patients. As shown in Table 1, at the time of data collection, the median time since the video’s upload was 136 days, with a publication time range of 7 to 1,712 days. The median number of likes per video is 133,013, the median number of shares is 47,205, the median number of comments is 4,838, and the median number of collections is 29,899. Among all MI-related short videos, the highest single video metrics were: likes (11,598,750), shares (1,844,375), collections (1,129,654), and comments (419,713).

**Table 1:** Characteristics of MI-Related videos on TikTok.

Parameters	TikTok (*N* = 270)
Likes	133,013 (0, 11,598,750)
Shares	47,205 (0, 1,844,375)
Collections	29,899 (0, 1,129,654)
Comments	4,838 (0, 419,713)
Followers	6,521,015 (3, 188,349,030)
Video duration (s)	132 (0, 762)
Time since video upload	136 (7, 1712)
Video source (*n*) (%)	
Healthcare Professionals	227 (84.1%)
Healthcare Institutions	7 (2.6%)
Media	19 (7.0%)
Individuals	14 (5.2%)
Social groups	1 (0.4%)
Medical Licensing Exams	2 (0.7%)
Traditional Chinese medicine (*n*) (%)	
Yes	37 (16.3%)
No	190 (83.7%)

**Figure 2:**
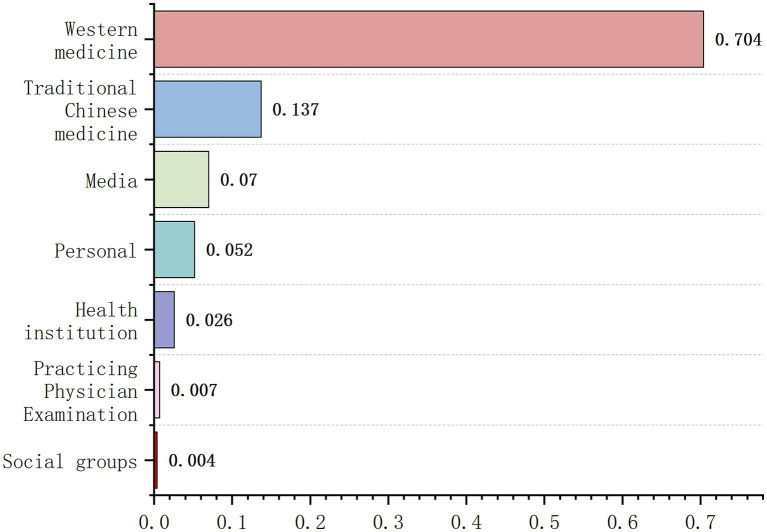
Video source analysis chart.

As shown in Figure 2, based on the source of video uploads, videos were primarily categorized into six groups: healthcare professionals, healthcare institutions, media outlets, individuals, social groups, and licensed physician examinations. Healthcare professionals accounted for the highest proportion at approximately 84.1% (227/270), followed by media outlets at about 7.0% (19/270). Among healthcare professionals, Western medicine practitioners accounted for 83.7% (190/227), while traditional Chinese medicine practitioners constituted 16.3% (37/227).

### 3.2 Video quality assessment

Descriptive analysis of GQS, JAMA and mDISCERN scoring scales revealed consistency in video content quality. As shown in Table 2, when evaluating video quality and reliability, the GQS scale yielded the highest proportion of videos scoring 3, accounting for 47.0% (127/270). The JAMA scale showed the highest proportion of videos scoring 2 (81.5%, 220/270). mDISCERN scores showed the highest proportion of videos rated 3 (45.9%, 124/270), followed by those rated 2 (31.5%, 85/270), with slightly fewer rated 4 (16.3%, 44/270). This indicates that TikTok short videos related to MI are relatively balanced in terms of assessment quality, timeliness, and effectiveness, generally ranking at a medium level. However, TikTok short videos related to MI exhibit certain variations across five dimensions: clarity, relevance, traceability, robustness, and impartiality.

**Table 2:** Analysis of GQS, JAMA, and mDiscern score sheet results.

Parameters	*N* (%)
GQS score	
1	13 (4.8%)
2	60 (22.2%)
3	127 (47.0%)
4	62 (23.0%)
5	8 (3.0%)
JAMA score	
0	0 (0.0%)
1	42 (15.6%)
2	220 (81.5%)
3	8 (3.0%)
4	0 (0.0%)
mDiscern score	
0	4 (1.5%)
1	13 (4.8%)
2	85 (31.5%)
3	124 (45.9%)
4	44 (16.3%)
5	0 (0.0%)

**Figure 3:**
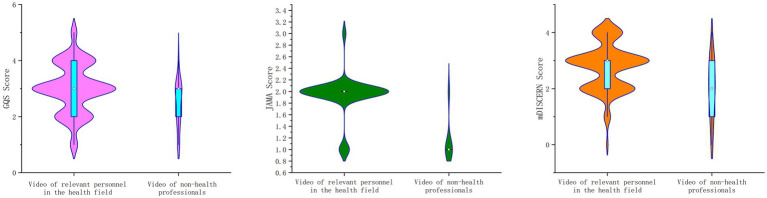
Comparison of GQS, JAMA, and mDISCERN scores between videos created by healthcare professionals and non-healthcare professionals.

Based on the source of the videos, we categorized them into videos featuring healthcare professionals and videos featuring non-healthcare professionals. Videos featuring healthcare professionals primarily included content from traditional Chinese medicine and Western medicine practitioners, medical institutions, Dingxiang Doctor, and medical professionals disseminating scientific knowledge in media news reports, totaling 250 relevant videos. Videos from non-healthcare professionals mainly encompass media reports on MI incidents, individual or team-produced content, and videos from medical licensing exam preparation platforms, totaling 20 videos. As shown in Table 3 and Figure 3, while non-healthcare professional videos exhibited certain differences compared to healthcare professional videos in terms of duration, likes, shares, collections, and comments, these differences were not statistically significant (*p* > 0.05). Regarding GQS scores, JAMA scores, and mDISCERN scores, videos posted by healthcare professionals received significantly higher ratings than those posted by non-healthcare professionals (*p* < 0.01).

**Table 3:** Comparison of videos by healthcare professionals and non-healthcare professionals.

Parameters	Healthcare professionals *N* = 250	Non-healthcare professionals *N* = 20	*p*
Runtime	89.5 (41, 186)	123.5 (59.5, 187)	0.245
Likes	10,405 (1,106, 54,973)	5470.5 (648.5, 37744.5)	0.486
Shares	3,174 (340, 21,377)	1,600 (384.5, 10,851)	0.486
Collections	3107.5 (482, 17,521)	1706.5 (148, 7311.5)	0.816
Comments	403 (62, 2032)	665 (138, 2,685)	0.486
GQS Score	3.01 ± 0.87	2.45 ± 0.83	0.005
JAMA Score	1.93 ± 0.36	1.15 ± 0.37	<0.001
mDISCERN Score	2.76 ± 0.80	2.00 ± 1.08	<0.001

Median and interquartile range are used to represent non-normally distributed continuous variables. Mean ± SD was used to represent normally distributed continuous variables.

### 3.3 Correlation analysis between video play metrics and content scores

**Figure 4:**
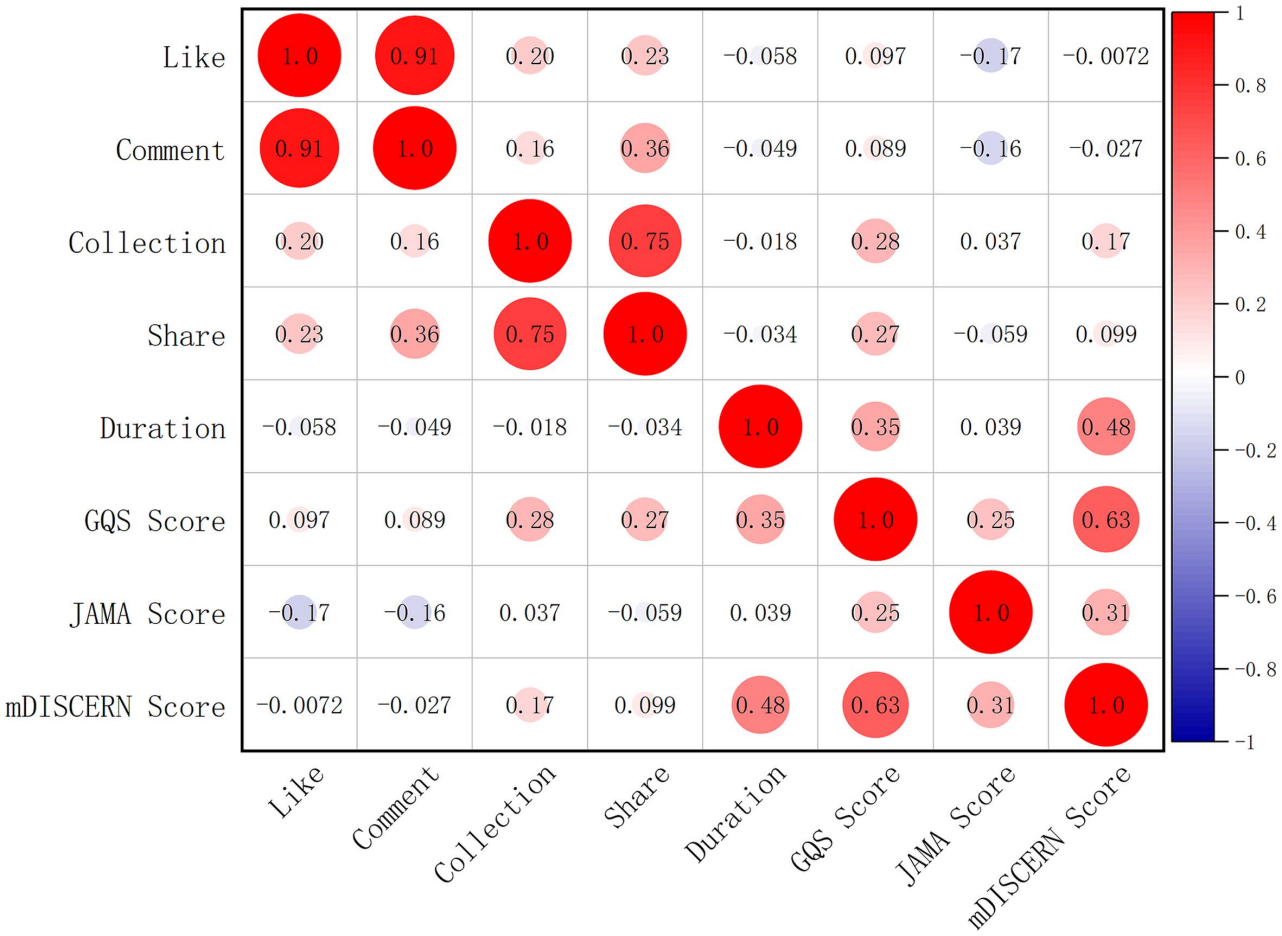
Heatmap of correlation between video play metrics and content scores.

As shown in Table 4 and Figure 4, correlation analysis was conducted between video play metrics and content quality scores. Results indicate significant correlations between GQS scores and the number of collections, shares, video duration, JAMA scores, and mDISCERN scores (*p* < 0.01), while no correlation was found with likes or comments (*p* > 0.05). JAMA scores showed significant correlations with likes, comments, GQS scores, and mDISCERN scores (*p* < 0.01), while exhibiting no correlations with bookmarks, shares, or video duration (*p* > 0.05). The mDISCERN score showed significant correlations with the number of collections, video duration, GQS score, and JAMA score (*p* < 0.01), but no correlations with the number of likes, comments, or shares (*p* > 0.05).

**Table 4:** Correlation analysis between video play metrics and video content quality scores.

Parameters	GQS score	JAMA score	mDISCERN score
Likes	0.110	0.005	0.907
Comments	0.142	0.008	0.657
Collections	<0.001	0.547	0.005
Shares	<0.001	0.335	0.105
Duration	<0.001	0.520	<0.001
GQS score		<0.001	<0.001
JAMA score	<0.001		<0.001
mDISCERN score	<0.001	<0.001	

### 3.4 Linear regression analysis of video scores and play metrics

As shown in Table 5, linear regression analysis was conducted between GQS scores, JAMA scores, mDISCERN scores, and the duration of the work, number of likes, number of comments, number of collections, and number of shares. Results indicate that video duration exhibits a significant positive correlation with both GQS and mDISCERN scores. Favorites count shows a positive correlation with both JAMA and mDISCERN scores. Conversely, likes and shares demonstrate a negative correlation with JAMA scores.

**Table 5:** Linear regression analysis of GQS, JAMA, and mDISCERN scores.

Parameters	GQS score		JAMA score		mDISCERN score	
	*β* value	*p*	*β* value	*p*	*β* value	*p*
Video duration	0.365	<0.001	0.025	0.680	0.485	<0.001
Likes	0.187	0.227	−0.374	0.029	0.062	0.681
Shares	0.210	0.047	−0.259	0.027	−0.010	0.923
Collections	0.119	0.225	0.272	0.012	0.189	0.048
Comments	−0.155	0.342	0.231	0.202	−0.085	0.592

## 4 Discussion

### 4.1 Discussion of key findings

As the world’s largest short video platform, TikTok features content spanning healthcare, wellness, nutrition, news, entertainment, and more (27–30). Particularly within China, rapid technological advancements in recent years have fostered growing reliance on this fast-paced, bite-sized approach to information consumption. Based on TikTok’s download volume and video views, it has become one of the primary channels for people to access information on healthcare, news, entertainment, and other topics (31). In recent years, as living standards have risen and life pressures have intensified, the incidence of MI has also increased, with many young patients even dying from sudden heart attacks (32–35). This has heightened public awareness of MI, driving increased consumption of related information through short videos (36). This study analyzed engagement metrics—including likes, shares, comments, collections, and video duration—for MI-related short videos on TikTok. Video quality was assessed using the GQS, JAMA, and mDISCERN scoring systems.

#### 4.1.1 Discussion on TikTok user engagement with MI-related short videos

Considering that engagement metrics (likes, shares, comments, collections) for videos uploaded within the past week may not yet be stable, we excluded newly uploaded MI short videos from the past week. Additionally, videos with irrelevant themes, invalid links, duplicates, or non-Mandarin content were excluded. After screening, 270 TikTok videos related to MI were obtained. These videos collectively received 35,913,507 likes (median: 133,013), 1,306,276 comments (median: 4,838), 8,072,837 collections (median: 29,899), and 12,745,256 shares (median: 47,205). This demonstrates that TikTok videos related to MI garnered extensive user engagement and participation. Through vivid presentations of videos, case studies, animations, audio, and text, these videos enable people to learn more intuitively and realistically about the symptoms, treatment methods, precautions, and effective prevention strategies for MI (37). They have now become an important channel for people to access medical and health-related knowledge.

#### 4.1.2 Discussion on video quality of MI-related short videos

The GQS score primarily reflects video quality (38), rated on a scale of 1 to 5. Among MI-related short videos, those scoring 3 were most prevalent, accounting for 47.0%. This indicates that key information was adequately addressed, with videos achieving moderate quality. The JAMA score primarily assesses whether videos include the publisher’s affiliation and credentials, reliable sources for content, disclosure of conflicts of interest, and publication dates. It evaluates the timeliness, validity, and compliance of video content, with a rating scale of 0–4 points (39). Among MI-related short videos, those scoring 2 on the JAMA scale were the most numerous, accounting for 81.5%. This indicates that the compliance of the vast majority of videos was at a moderate level. The mDISCERN scoring scale primarily reflects five aspects of the video: clarity, relevance, traceability, robustness, and fairness, with a rating scale of 1 to 5 (38, 40). Videos scoring 3 were the most numerous, accounting for 45.9%, followed by those scoring 2 at 31.5%, while those scoring 4 were slightly fewer at 16.3%. This indicates that a significant proportion of videos fall within the moderate range for content quality.

This study employed the GQS, JAMA, and mDISCERN scales to evaluate short videos. These tools measure the “educational quality” of short video information—specifically structural features such as author transparency, source traceability, clarity of expression, and balanced presentation of treatment options—rather than “clinical accuracy” or “guideline adherence.” Within this study’s sample, moderate or low GQS, JAMA, and mDISCERN scores do not imply the presence of misinformation. Rather, they reflect inherent limitations of the short video format, such as failure to cite valid sources, lack of discussion on treatment risks or alternatives, or fragmented information presentation due to format constraints.

In summary, the overall video quality of TikTok short videos related to MI remains at a moderate level. These videos still exhibit certain shortcomings in terms of comprehensiveness, compliance, effectiveness, relevance, robustness, and clarity. It is recommended that future uploaders enhance video quality and reliability by extending video duration to provide more comprehensive explanations of MI, citing valid references and sources, and disclosing their professional credentials.

#### 4.1.3 Comparative analysis and discussion of sources for MI-related short videos

It is noteworthy that sources of MI short videos on TikTok exhibit certain variations. While such content primarily originates from healthcare professionals—particularly Western medicine practitioners—a portion also stems from ordinary users, media outlets, or social organizations. Drawing from previous studies on platforms like YouTube, this phenomenon is largely tied to the content themes and platform mechanisms. Highly specialized diseases like MI are predominantly covered by medical creators, while simpler health issues attract significant participation from ordinary users (41, 42). Unlike open platforms such as YouTube, the Chinese version of TikTok enforces a strict physician verification system. Only users verified through official accounts of top-tier hospitals or as licensed physicians can publish medical science content. Unverified users posting about serious medical topics like MI may face higher review thresholds and require more specialized medical knowledge. Furthermore, MI is a time-sensitive emergency with high out-of-hospital mortality and low public awareness. Unlike everyday health topics, non-professional users often lack understanding of the pathological mechanisms and emergency measures for acute MI. This makes them more prone to sparking controversy or being flagged by the platform for disseminating misleading information when posting related content. This objectively dampens the motivation for patients or ordinary users to create content, leading to a more concentrated voice of healthcare professionals on this topic.

Comparing videos created by healthcare professionals with those by non-healthcare professionals revealed no statistically significant differences in likes, comments, collections, shares, or video duration. However, videos created by healthcare professionals received significantly higher scores on the GQS, JAMA, and mDISSCERN assessments compared to those created by non-healthcare professionals. This indicates that videos created by healthcare professionals demonstrate significantly superior content quality, comprehensiveness, effectiveness, relevance, and scientific rigor compared to those produced by non-healthcare professionals. Considering these findings comprehensively, two potential reasons emerge: First, platform mechanisms may incentivize traffic-driven content, prompting some non-professionals to create attention-grabbing videos to garner more likes, comments, collections, and shares (43). Second, compared to healthcare professionals, firsthand accounts of medical experiences by MI patients often resonate more emotionally, eliciting greater empathy and attention from general audiences (44). Although videos posted by MI patients reflect genuine personal experiences, they often lack scientific rigor and comprehensiveness, presenting only partial perspectives or anecdotal summaries. Given the lower quality of non-healthcare professional videos, platforms should strengthen content review for future MI-related short videos to prevent the dissemination of misinformation that could mislead other patients about MI.

### 4.2 Correlation analysis between user engagement metrics for video playback and video quality

To explore the relationship between video engagement metrics and video quality, we conducted correlation and linear regression analyses. Results indicate that video duration exhibits a significant positive correlation with both GQS scores and mDISCERN scores. Similarly, the number of collections correlates positively with both JAMA scores and mDISCERN scores. Conversely, the number of likes and shares shows a negative correlation with JAMA scores. This indicates that longer videos with higher collection counts demonstrate superior quality and reliability. Conversely, videos with high like and share counts, while garnering viewer favor and substantial traffic, often lack disclosure of creator, attribution, and copyright information. Such videos exhibit lower quality and certain non-compliance issues. This may stem from video publishers’ self-protection instincts, as they are reluctant to reveal professional credentials, copyright details, or funding sources. Comment volume showed no significant linear regression relationship with GQS scores, JAMA scores, or mDISCERN scores, indicating no clear correlation between comment count and video quality. The data analysis indicates that for medical science communication videos on MI, enhanced validity and compliance reviews are essential. This includes improving disclosure of author/organization details, relevant certifications, ownership, and copyright information to ensure videos comply with regulations. Additionally, extending video duration is recommended to present more comprehensive content on MI, elevate video quality, and provide patients with thorough information—rather than prioritizing sensationalism and traffic acquisition.

### 4.3 Impact on public health communication

With the rise of short video platforms, social media like TikTok has become a primary channel for health information access. While these videos enhance public health awareness, they also pose challenges to information reliability, necessitating multi-stakeholder collaboration (45). From a positive perspective, as a critical emergency condition, the proliferation of short videos has significantly heightened public attention toward cardiovascular health (46). Through vivid visual presentations and accessible explanations, this content rapidly conveys typical symptoms and emergency measures for MI, helping individuals recognize early warning signs and take timely action (47). For younger audiences in particular, this accessible format breaks down barriers to traditional health education, bolstering preventive awareness. Furthermore, videos created by medical professionals often emphasize managing risk factors associated with MI, promoting healthier lifestyles that may reduce disease incidence over the long term.

However, the reliability of such videos poses risks to public health. Research indicates that due to their brevity and incomplete explanations, many short videos suffer from fragmented, biased, or even erroneous information—such as exaggerating the efficacy of certain therapies or disregarding personalized medical principles (48). Public reliance on these unverified sources may lead to delayed diagnosis, misdiagnosis, risks of self-medication, or unnecessary anxiety (49, 50). More critically, the dissemination of false information intertwined with advertisements may erode trust in formal healthcare systems and undermine effective doctor-patient communication (51, 52). To maximize the public health benefits of short videos, platforms must strengthen content moderation, promote official accounts from authoritative medical institutions, and utilize algorithms to reasonably extend video duration while prioritizing high-quality content from healthcare professionals. Healthcare institutions should proactively engage by creating scientifically sound and engaging content while conducting public media literacy education to help users critically evaluate health information. Through multi-stakeholder collaboration, short videos can become effective tools for promoting cardiovascular health rather than sources of risk.

## 5 Conclusion

This study collected 270 short videos related to MI on TikTok, which garnered numerous likes, collections, shares, and comments. Video creators included healthcare professionals, media outlets, MI patients, and support groups, with healthcare professionals predominantly representing Western medicine. All videos were individually reviewed and scored using the GQS, JAMA, and mDISCERN assessment tools, revealing that the overall quality and reliability of MI-related short videos were moderate. Correlation analysis between the scoring results and engagement metrics (likes, collections, etc.) indicated that video quality was closely associated with video duration and the number of collections. Based on the scoring scales and our viewing impressions, we attribute the low video quality primarily to their brevity. Most videos focus on a single aspect—such as triggers, symptoms, pathogenesis, treatment methods, or prognosis—failing to provide comprehensive coverage of MI. Additionally, video creators often omit references, sources, and relevant certifications or copyright information for their content. MI is characterized by acute onset, high mortality, treatment challenges, and poor prognosis. Understanding its causative factors and symptoms enhances public awareness and prevention capabilities (53–55). In summary, we recommend that short videos on MI appropriately increase their duration and incorporate credible references or valid supporting evidence within the content. This will enhance the comprehensiveness and scientific rigor of the material, thereby improving the quality and reliability of such videos. Such practices also provide an effective basis for enhancing the quality of other medical-related videos.

## 6 Strengths and limitations of the study

### 6.1 Strengths of the study

This study represents the first systematic evaluation of the quality and reliability of short videos about MI on TikTok, holding significant practical implications. Its strengths are primarily threefold: First, the study focuses on one of the world’s most popular short video platforms, directly addressing the primary channel through which the public—especially younger demographics—access health information today. Consequently, the findings carry substantial public health reference value. Second, the cross-sectional design employed rigorous video screening and scientific evaluation tools, ensuring systematic methodology and objective outcomes that establish a methodological foundation for future research. Third, the study not only quantified information quality but also analyzed how content sources and types influence reliability, clearly identifying core deficiencies and potential risks in existing platform information. This provides content creators and platform regulators with explicit directions for improvement.

### 6.2 Research limitations

Due to platform constraints and human evaluation factors, this study has limitations. First, it reflects information conditions only at a specific point in time. Given TikTok’s rapid content updates and dynamic information landscape, the long-term validity of its conclusions requires further observation. Second, the research sample may not comprehensively cover all relevant videos on the platform. Search results are influenced by personalized factors such as user location, potentially introducing selection bias. Third, while the evaluation scales used are validated, they may not fully accommodate the unique dissemination patterns of short videos characterized by high information density and fragmented formats. Finally, the study focuses on content quality itself without assessing the actual impact of this information on viewers’ knowledge, attitudes, and health behaviors, leaving its true public health effects unclear.

Future research could collect samples at multiple time points, continuously analyzing the same batch of popular videos or content under the same topic tags to examine the evolution of health information on short video platforms. This approach would reveal more comprehensive insights into the dynamic characteristics of the platform’s content ecosystem. Given that scales like GQS, JAMA, and mDISCERN were not designed for short video formats, a new evaluation framework incorporating dimensions such as information accuracy and visual presentation should be developed based on the unique characteristics of short video communication—such as the integration of audio and visuals and time constraints—and subjected to systematic reliability and validity testing. Future research should integrate content analysis with audience studies. For instance, through surveys and analysis of viewer comments, we can explore the actual impact of short video health information on public cognitive biases, risk perceptions, healthcare-seeking behaviors, and self-management practices, thereby better evaluating the effectiveness of public health communication.

## Data Availability

The original contributions presented in the study are included in the article/Supplementary material, further inquiries can be directed to the corresponding authors.

## Supplementary material

SUPPLEMENTARY FIlE 1 Detailed information and scoring results of myocardial infarction-related short videos.

SUPPLEMENTARY FILE 2 GQS scoring scale.

SUPPLEMENTARY FILE 3 JAMA scoring scale.

SUPPLEMENTARY FILE 4 mDISCERN scoring scale.
