# Supplementary material for: The quality and reliability of short videos about myocardial infarction on TikTok: a cross-sectional study
Source: Front Public Health. 2026 Mar 12;14:1751884. doi: 10.3389/fpubh.2026.1751884 (PMC13018143; doi:10.3389/fpubh.2026.1751884)
Supplement: SUPPLEMENTARY FILE 3 — JAMA scoring scale. [file Table_3.DOCX]

**JAMA**

**Table S2. The Journal of the American Medical Association (JAMA) benchmark criteria.**

| **Criteria** | **Description** |
| --- | --- |
| Authorship | Authors and contributors, their affiliations, and relevant credentials should be provided.  1 |
| Attribution | References or sources for all content should be listed clearly, and all relevant copyright information noted.  1 |
| Currency | Website ownership should be prominently and fully disclosed, as should any sponsorship, advertising, underwriting, commercial funding arrangements or support, or potential conflicts of interest.  1 |
| Disclosure | Dates that content was posted and updated should be indicated.  1 |
