# Supplementary material for: The quality and reliability of short videos about myocardial infarction on TikTok: a cross-sectional study
Source: Front Public Health. 2026 Mar 12;14:1751884. doi: 10.3389/fpubh.2026.1751884 (PMC13018143; doi:10.3389/fpubh.2026.1751884)
Supplement: SUPPLEMENTARY FILE 4 — mDISCERN scoring scale. [file Table_4.DOCX]

**mDISCERN**

Supplementary: Modified DISCERN quality criteria for assessing the reliability of video. (1 point for answer ‘yes’, 0 point for answer ‘no’)

Reliability Score

1. Is the video clear, concise, and understandable?

2. Are valid sources cited?

3. Is the content presented balanced and unbiased?

4. Are additional sources of content listed for patient reference?

5. Are areas of uncertainty mentioned?
